# A large pericardial cystic lymphangioma presenting as acute-onset respiratory distress in a child: a case report

**DOI:** 10.1186/s13256-022-03576-4

**Published:** 2022-10-31

**Authors:** Hashan Pathiraja, Dhammike Rasnayake, Thilini Muthukumarana, Channa de Silva, Wasantha Sathkorala, Sandini Gunaratne, Shaman Rajindrajith, Sachith Mettananda

**Affiliations:** 1 Colombo North Teaching Hospital, Ragama, Sri Lanka; 2 National Hospital for Respiratory Diseases, Welisara, Sri Lanka; 3 Lady Ridgeway Children’s Hospital, Colombo, Sri Lanka; 4 Department of Paediatrics, University of Colombo, Colombo, Sri Lanka; 5 Department of Paediatrics, Faculty of Medicine, University of Kelaniya, Thalagolla Road, Ragama, 11010 Sri Lanka

**Keywords:** Lymphangioma, Pericardial, Mediastinal sift

## Abstract

**Background:**

Lymphangiomas are rare benign malformations of the lymphatics that occur due to blockage of the lymphatic system during fetal development. They commonly occur in the neck and axilla, while involvement of the pericardium is rare. We report herein the case of a 16-month-old Sri Lankan child with a large pericardial cystic lymphangioma presenting with sudden-onset shortness of breath.

**Case presentation:**

A 16-month-old Sri Lankan boy presented with sudden-onset dyspnea for 1-day duration following a febrile illness that lasted 2 days. On examination, he was afebrile and had subcostal, intercostal, and suprasternal recessions, with a respiratory rate of 50 breaths per minute. He had a loud expiratory grunt. The chest expansion was reduced on the right side, which was dull to percussion. Auscultation revealed a marked reduction of air entry over the right lower and mid zones. Chest X-ray showed a well-demarcated opacity involving the lower and mid zones of the right hemithorax associated with a tracheal shift to the opposite side. Ultrasound scan of the chest revealed fluid-filled right hemithorax suggesting a septate pleural effusion. A contrast-enhanced computed tomography scan of the thorax showed a large multiloculated extrapulmonary cystic lesion involving the right hemithorax with a mediastinal shift towards the left side associated with displacement of the right-side mediastinal structures. He underwent mini-thoracotomy and surgical excision of the cyst. A large cyst originating from the pericardium was observed and excised during surgery. Histological examination revealed a lesion composed of cysts devoid of a lining epithelium but separated by connective tissue, mature adipose tissue, and lymphoid aggregates. The child showed complete recovery postoperatively with full expansion of the ipsilateral lung.

**Conclusion:**

We report the case of a patient with cystic lymphangioma who was perfectly well and asymptomatic until 16 months of age. This case report presents the very rare occurrence of a large cystic lymphangioma originating from the pericardium. It highlights the importance of considering rare possibilities and performing prompt imaging in situations of diagnostic uncertainty to arrive at an accurate diagnosis that can be lifesaving.

## Background

Lymphangiomas are rare benign malformations of the lymphatics that occur due to blockage of the lymphatic system during fetal development [1]. They are characterized by abnormal proliferation of lymphatic vessels and failure of communication between the primitive lymphatic sacs and the venous system [2]. Lymphangiomas can occur anywhere in the body; however, they involve the neck and axilla commonly [3]. Involvement of the pericardium is extremely rare. We report herein the case of a 16-month-old Sri Lankan child with a large pericardial cystic lymphangioma presenting with sudden-onset shortness of breath.

## Case presentation

A 16-month-old Sri Lankan boy presented with sudden-onset difficulty in breathing for 1-day duration. He had a febrile illness with coryzal symptoms for 3 days, which had resolved 2 days prior to this presentation. There was no history of foreign-body aspiration or trauma. He was born to non-consanguineous parents at term and had an uneventful neonatal period. He did not have any previous hospital admissions or recurrent wheezing episodes and was developmentally normal.

On examination, the child was afebrile and not pale or cyanosed. His weight was 8.8 kg (between −1SD and −2SD), and his length was 82 cm (between median and +1SD). He had moderate respiratory distress with subcostal, intercostal, and suprasternal recessions, and his respiratory rate was 50 breaths per minute. He had a loud expiratory grunt. The chest expansion was reduced on the right side, which was dull to percussion. Auscultation revealed a marked reduction of air entry over the lower and mid zones of the right chest; there were no added sounds. His arterial oxygen saturation was 98% in room air. Cardiovascular examination revealed a pulse rate of 140 per minute and blood pressure of 94/60 mmHg. The apex was felt at the fifth intercostal space lateral to the midclavicular line, and his heart sounds were normal. The rest of the clinical examination was normal.

**Fig. 1:**
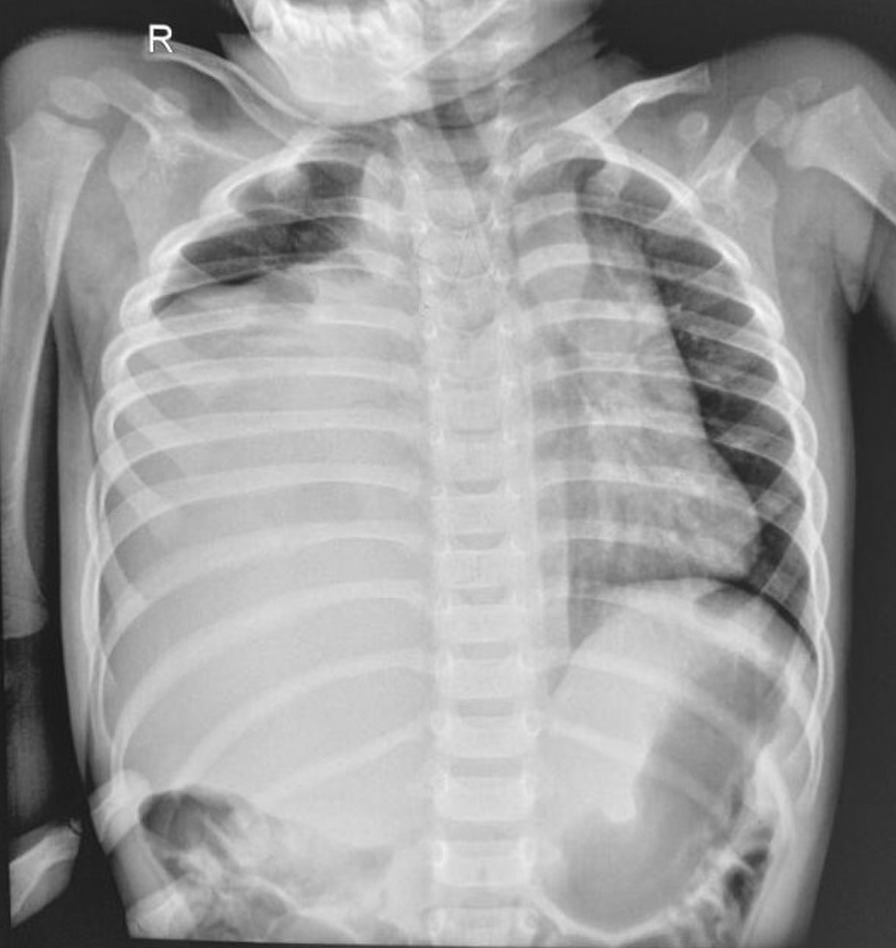
Chest X-ray showing well-demarcated opacity in the lower and mid zones of the right hemithorax with shifting of the trachea to the left side

Urgent chest X-ray performed due to lateralizing physical signs showed a well-demarcated opacity involving the lower and mid zones of the right hemithorax associated with a tracheal shift to the opposite side (Fig. 1). The right heart and diaphragmatic borders were obliterated. Ultrasound scan of the chest revealed fluid-filled right hemithorax suggesting a septate pleural effusion. His full blood count revealed white cell count of 17,000/mm^3^ (neutrophils 7300/mm^3^, lymphocytes 8100/mm^3^, eosinophils 300/mm^3^), hemoglobin of 7.3 g/dL, and platelet count of 607,000/mm^3^. His C-reactive protein was 10.5 mg/L. His serum electrolytes and renal and liver function tests were normal. He was treated for a possible right lower lobe pneumonia with parapneumonic pleural effusion with intravenous cefotaxime 50 mg/kg 8 hourly.

**Fig. 2:**
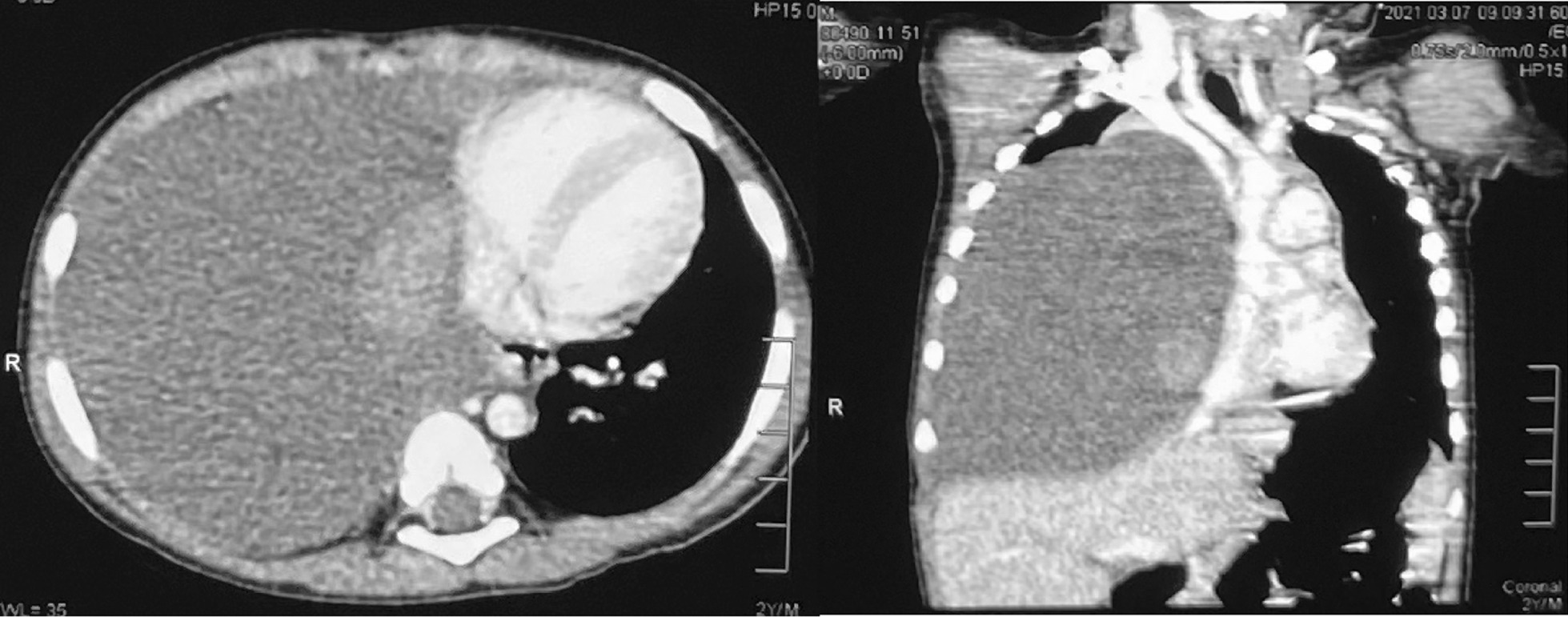
Contrast-enhanced CT scan of the chest showing a large multiloculated extrapulmonary cystic lesion (measuring 11.5 cm × 11.3 cm × 9.5 cm) involving the right hemithorax with a mediastinal shift towards the left and mass effect and displacement of the right-side mediastinal structures. The right lung is compressed superiorly, and the right main pulmonary artery is narrowed

Due to the absence of fever, negative inflammatory markers, and the unusual nature of the chest X-ray, which led to diagnostic uncertainty, an urgent contrast-enhanced CT scan of the thorax was performed on the following day (Fig. 2). This showed a large multiloculated extrapulmonary cystic lesion involving the right hemithorax with a mediastinal shift towards the left side. This was associated with the displacement of the right-side mediastinal structures, including superior and inferior vena cava, narrowing of the right main pulmonary artery, and compression of the right lung superiorly. The lung parenchyma was normal. The CT appearance was in favor of a benign cystic lesion in the mediastinum, most likely a lymphangioma. The echocardiography was normal.

**Fig. 3:**
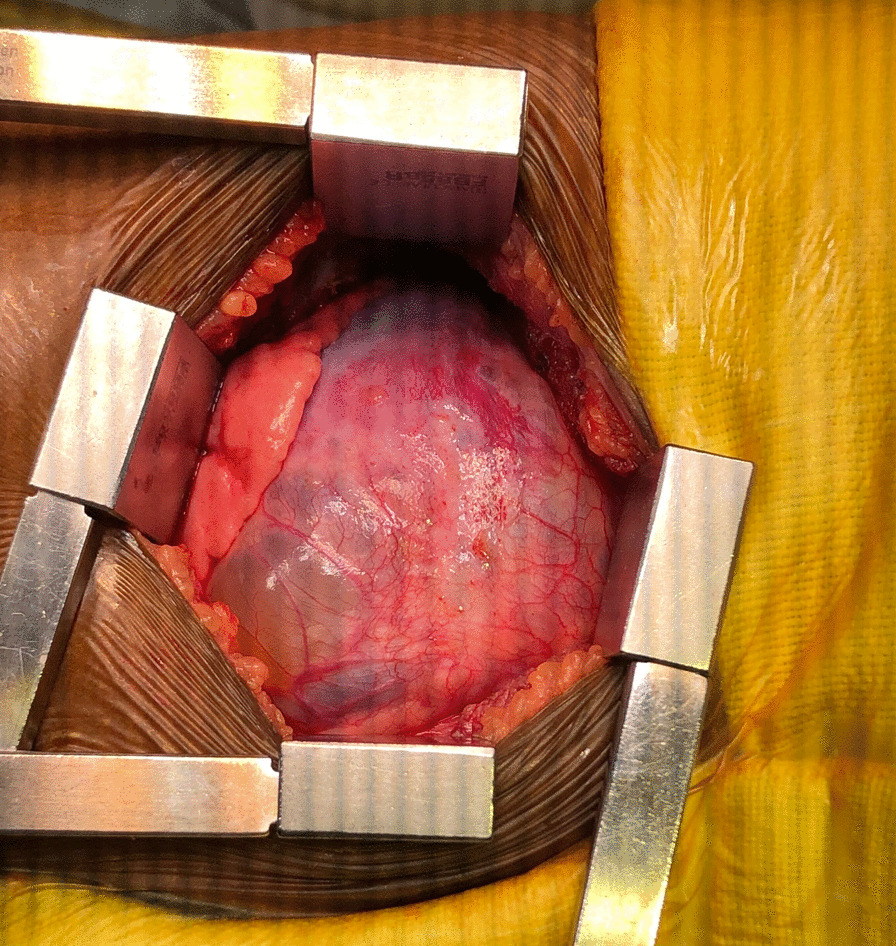
Intraoperative photograph of the cyst originating from the pericardium

**Fig. 4:**
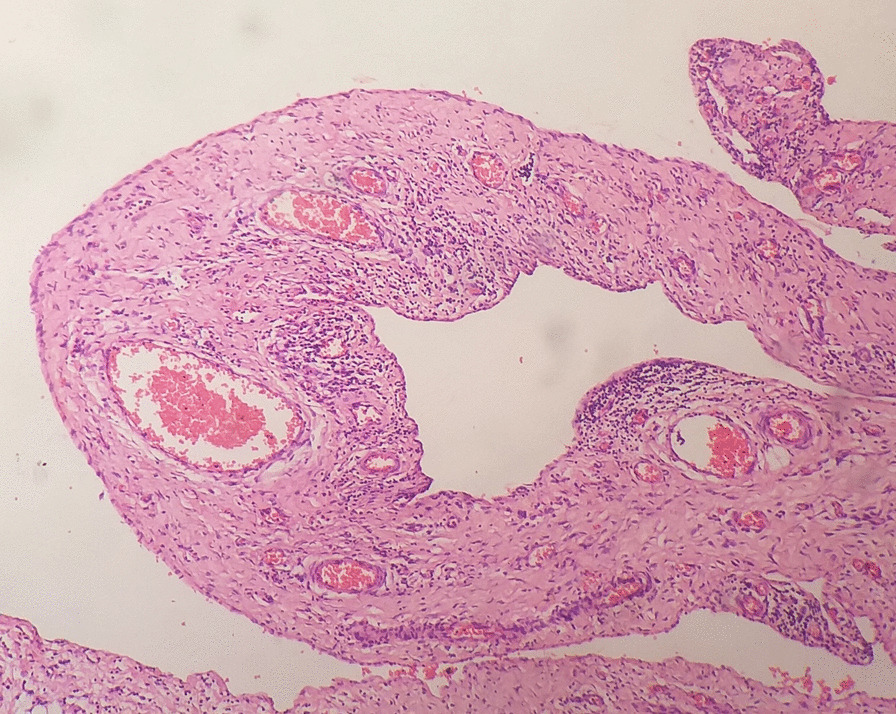
Microscopy of a dilated lymphatic channel with surrounding lymphocytes (H and E stain)

**Fig. 5:**
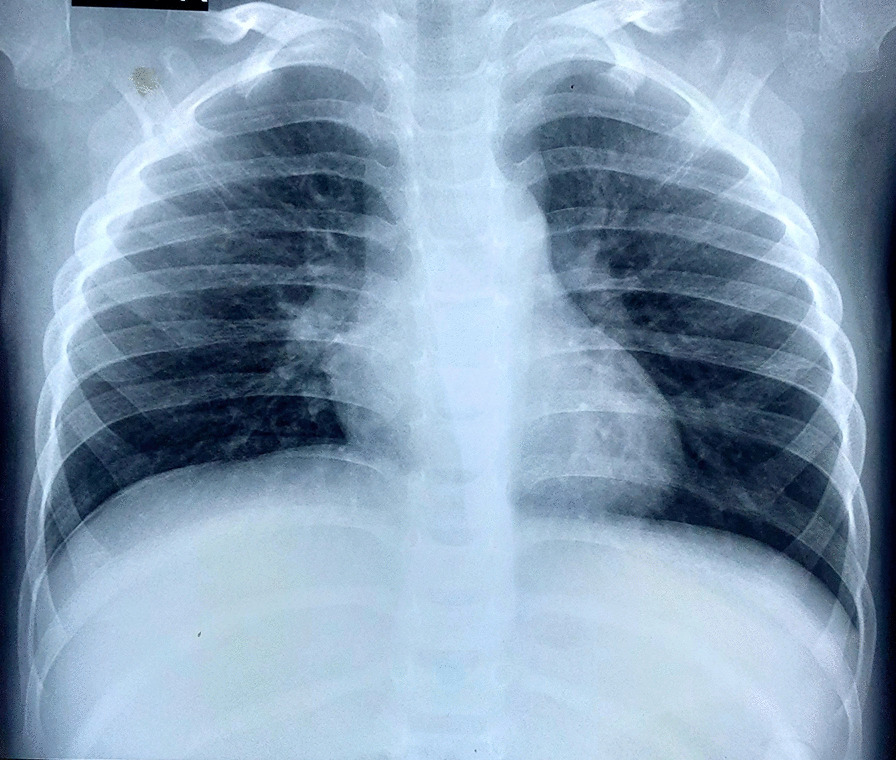
Chest X-ray taken on the 10th postoperative day, showing complete resolution and expansion of the right lung

He initially underwent ultrasound-guided aspiration of cyst fluid for immediate relief of respiratory distress. Analysis of aspiration fluid revealed clusters of pulmonary macrophages and lymphocytes in the background of blood with necrotic material and no malignant cells. However, the response to aspiration was short-lasting due to re-accumulation of cyst fluid. A week later, he underwent a mini-thoracotomy and surgical excision of the cyst. During surgery, he was intubated with a size 4.5 endotracheal tube and provided pressure-controlled ventilation with peak inspiratory pressure of 18, peak end-expiratory pressure of 6, and respiratory rate of 25. A large cyst measuring 8 cm × 6 cm × 1.5 cm originating from the pericardium was observed during surgery and was completely excised (Fig. 3). The histological examination revealed a lesion composed of cysts devoid of a lining epithelium but separated by connective tissue with mature adipose tissue and lymphoid aggregates. There was evidence of chronic inflammation but no evidence of malignancy (Fig. 4). On the basis of the histological findings, the diagnosis of cystic lymphangioma was confirmed. The child showed complete recovery postoperatively with full expansion of the ipsilateral lung (Fig. 5). He remained symptom free at 6-week and 3-month follow-up visits.

## Discussion

Lymphangiomas are rare malformations of the lymphatic system due to abnormalities in fetal development [4]. Although the majority are present and diagnosed at birth, some lymphangiomas present later in life [5]. Here, we report a patient with cystic lymphangioma who was perfectly well and asymptomatic until 16 months of age.

Our patient presented with sudden-onset difficulty in breathing, and his chest X-ray showed opacity involving the lower and mid zones of the right hemithorax. He was initially diagnosed as having lobar pneumonia with parapneumonic effusion. However, as the clinical picture did not resemble severe pneumonia and the initial inflammatory markers were negative, a contrast-enhanced CT scan was performed without delay. The diagnosis of lymphangioma was suggested only by the CT scan, which showed an extrapulmonary cystic lesion. In addition to helping to make the diagnosis, the CT scan was crucial for the configuration of the operative plan.

Other differential diagnoses for a cystic mass in the thorax include cystic hygroma and hemangioma. Cystic hygroma is also a type of lymphatic malformation that is characterized by large, interconnected lymphatic cysts lined by a thin endothelium. However, cystic hygromas are usually present at birth, covered by skin, and mostly located in the cervical region [6]. Deep hemangioma of the lung was also a possibility; however, the absence of contrast enhancement in the CT excluded that diagnosis [7].

Late presentation of pericardial cystic lymphangiomas at 16 months of age with sudden-onset respiratory distress is the most unusual feature of this case report. Although late presentations of pericardial cystic lymphangiomas have been reported in literature, the common presentations include chest pain, cough, palpitations, gradual-onset dyspnea, and chylopericardium [8–10]. A recent review of 35 cases of cardiac or pericardiac cystic lymphangioma revealed that eight patients were asymptomatic and were diagnosed incidentally. The sudden appearance of symptoms of lymphangiomas is generally attributed to rapid enlargement of an existing lesion due to inflammation, hemorrhage, or trauma [11]. In our patient, the sudden onset of respiratory distress was most likely due to an intracystic hemorrhage.

The child underwent complete surgical excision of a cystic lymphangioma with complete resolution. Although this is the treatment of choice, surgery may sometimes not be feasible due to the infiltrative nature of lesions. Intralesional injection of sclerosing agents such as OK432 (picibanil) or 100% ethanol, localized laser therapy, and systemic therapy with propranolol or sirolimus are other treatment options in patients who are not amenable or responsive to surgery [3].

## Conclusion

We report a case with the rare occurrence of a large cystic lymphangioma originating from the pericardium. It highlights the importance of considering rare possibilities and performing prompt imaging in situations of diagnostic uncertainty to arrive at an accurate diagnosis that can be lifesaving.

## Data Availability

Not applicable.

## Abbreviation

CT: Computed tomography

## References

[1] Kennedy TL, Whitaker M, Pellitteri P, Wood WE. Cystic hygroma/lymphangioma: a rational approach to management. Laryngoscope. 2001;111(11 Pt 1):1929–37. 10.1097/00005537-200111000-00011 11801972

[2] Yokoigawa N, Okuno M, Kwon AH. Cystic lymphangioma of the chest wall: a case report. Case Rep Gastroenterol. 2014;8(3):393–7. 10.1159/000370030 PMC4296252 25606029

[3] Lee WS, Kim YH, Chee HK, Lee SA, Kim JD, Kim DC. Cavernous lymphangioma arising in the chest wall 19 years after excision of a cystic hygroma. Korean J Thorac Cardiovasc Surg. 2011;44(5):380–2. 10.5090/kjtcs.2011.44.5.380 PMC3249346 22263194

[4] Faul JL, Berry GJ, Colby TV, Ruoss SJ, Walter MB, Rosen GD, Raffin TA. Thoracic lymphangiomas, lymphangiectasis, lymphangiomatosis, and lymphatic dysplasia syndrome. Am J Respir Crit Care Med. 2000;161(3 Pt 1):1037–46. 10.1164/ajrccm.161.3.9904056 10712360

[5] Karkos PD, Spencer MG, Lee M, Hamid BN. Cervical cystic hygroma/lymphangioma: an acquired idiopathic late presentation. J Laryngol Otol. 2005;119(7):561–3. 10.1258/0022215054352144 16175984

[6] Mirza B, Ijaz L, Saleem M, Sharif M, Sheikh A. Cystic hygroma: an overview. J Cutan Aesthet Surg. 2010;3(3):139–44. 10.4103/0974-2077.74488 PMC3047730 21430825

[7] Maruyama M, Isokawa O, Hoshiyama K, Hoshiyama A, Hoshiyama M, Hoshiyama Y. Diagnosis and management of giant hepatic hemangioma: the usefulness of contrast-enhanced ultrasonography. Int J Hepatol. 2013;2013: 802180. 10.1155/2013/802180 PMC3670574 23762570

[8] Cailleba L, Labrousse L, Marty M, Montaudon M, Gerbaud E. Pericardial cystic lymphangioma. Eur Heart J Cardiovasc Imaging. 2013;14(3):246. 10.1093/ehjci/jes202 23034994

[9] Vinayakumar D, Arunkumar G, Sajeev CG, Rajesh G, Muneer K, Haridasan V, Babu K, Krishnan MN. Cystic lymphangioma of pericardium presenting as isolated chylopericardium—a case report. Indian Heart J. 2014;66(1):119–21. 10.1016/j.ihj.2013.12.020 PMC4054826 24581109

[10] Pichler Sekulic S, Sekulic M. Primary cardiac and pericardial lymphangiomas: clinical, radiologic, and pathologic characterization derived from an institutional series and review of the literature. Virchows Arch. 2022;480(6):1211–21. 10.1007/s00428-022-03269-9 35013774

[11] Mehrnahad M, Kord A, Rezaei Z, Kord R. Late diagnosis of generalized lymphangiomatosis in a woman presenting with respiratory distress. Radiol Case Rep. 2020;15(8):1189–93. 10.1016/j.radcr.2020.05.021 PMC7292890 32550956

